# Editorial: Women pioneering neuroinformatics and neuroscience-related machine learning, 2024

**DOI:** 10.3389/fninf.2025.1724386

**Published:** 2025-11-12

**Authors:** Rositsa Paunova, Alice Geminiani

**Affiliations:** 1 Department of Psychiatry and Medical Psychology, Medical University Plovdiv, Plovdiv, Bulgaria; 2 Research Institute and SRIPD-MUP, Translational and Computational Neuroscience Group, Medical University Plovdiv, Plovdiv, Bulgaria; 3 Department of Brain and Behavioral Sciences, University of Pavia, Pavia, Italy

**Keywords:** neuroscience, brain medicine, artificial intelligence, machine learning, neuroinformatics, *in silico* neuroscience

## Introduction

In recent years, neurosciences have undergone a translational turn (Kapur et al., 2012), as computational tools from AI, neuroinformatics and machine learning fields are increasingly employed within biological and clinical studies on neurological and psychiatric conditions (Insel, 2014). In this effort, despite their pivotal contributions, women remain underrepresented in neuroinformatics and neuroscience-focused machine learning, underscoring the need for greater equity and visibility (UNESCO, 2024).

In this Research Topic, we highlight studies that exemplify the translational shift of computational neurosciences and highlight the impact of women in this field. The featured papers showcase applications of AI, neuroinformatics, and computational modeling in brain research and medicine, spanning diverse domains from neural signal processing to clinical neuroscience, thus integrating the experimental and social dimensions of brain disorders into a data-driven and epistemically robust framework (Friston et al., 2014), based on rigorous biological and computational measures (He et al., 2020). By emphasizing studies with women as main or leading authors, this Research Topic aims to increase the visibility of their scientific contributions, promote gender equity, and inspire future researchers in these rapidly evolving fields.

## Contributions in this Research Topic

### AI for brain medicine

Alonso-Vázquez et al. address the challenge of imagined speech decoding using EEG signals. Their study demonstrated that training models on both pronounced and imagined speech improves the decoding accuracy for imagined speech alone, allowing more participants to reach thresholds for functional communication.

They illustrate how multi-condition training paradigms can help preserve communicative agency in individuals with motor neuron diseases (Birbaumer et al., 2014). By integrating overt data before speech is irretrievably lost, the study shows that BCIs can be embedded in the trajectory of disease progression, creating a continuum between residual function and technological support. This underscores how translational neurotechnology can mediate between clinical deterioration and the preservation of subjectivity.

AlHarkan et al. focus on the growing global burden of dementia and related disorders. Their study demonstrates that machine learning models trained on accessible demographic and clinical features can achieve predictive accuracies exceeding 90%. Crucially, these models do not rely on costly imaging or invasive biomarkers, but point toward the possibility of scalable, low-cost screening tools (Livingston et al., 2020).

Their work applies a nomothetic network approach, in which population-level models identify risk patterns that can be adapted to individuals (Borsboom, 2017). The inclusion of gender-specific models reflects the importance of personalization, acknowledging that both biological and sociocultural dimensions shape trajectories of cognitive decline, and also underscoring the ethical imperative of equity in translational neuroscience (Vollmann, 2018).

Aldrees et al. present an explainable AI framework for predicting autism spectrum disorder in toddlers. By curating diverse datasets and applying advanced feature selection, they achieved accuracies of up to 99% with optimized XGBoost models. Yet the study's significance lies not merely in predictive accuracy: it extends into the interpretive and practical realm, linking outputs to tailored educational strategies.

This dual focus exemplifies the epistemic ambition of explainable AI in medicine (Doshi-Velez and Kim, 2017)—not only to predict, but also to render intelligible the biological and behavioral signatures of complex conditions. By embedding AI in the educational trajectories of children with ASD, the work bridges clinical prediction with social application, thus expanding the horizon of what translational neuroscience can achieve.

### Neuroinformatics for large-scale brain datasets

Baazaoui et al. introduce MAGIC, a multisite clinical and imaging repository for acute ischemic stroke, consolidating de-identified radiological and structured clinical data from multiple European stroke centers to facilitate collaborative research. They outline the technical, legal, and ethical framework for the repository and describe its planned applications, including machine learning–based outcome prediction and imaging model development, showing how neuroinformatics and machine learning can be integrated with clinical studies.

Valevicius et al. also present a hey integrate the PhysIO MATLAB toolbox into the web-based CBRAIN platform, enabling remote, scalable preprocessing of physiological noise in fMRI with a user-friendly GUI, provenance tracking, and BIDS support. Their benchmarking shows substantially reduced user setup time and improved workflow efficiency for large datasets, reinforcing CBRAIN's potential as an open neuroscience infrastructure for multimodal, large-scale neuroimaging.

### *In silico* investigation of neural and brain data

Fabbri et al. present Digitoids, a modular *in silico* platform that integrates Hodgkin-Huxley–type neuronal dynamics with oxygen diffusion and consumption models to simulate network electrophysiology under varying oxygen conditions. Their simulations reproduce experimentally observed mean firing rates in neuronal cultures, and predict how oxygen limitation reduces firing and synchrony—thus highlighting the importance of including bioenergetic constraints in computational models of neural activity. Their work is an example of applying computational neuroscience tools to advance experimental neuroscience.

Chan et al. introduce a novel approach to generating *in silico* diffusion-weighted imaging (DWI) scalar maps, directly from fluid-attenuated inversion recovery MRI volumes using generative adversarial networks (GANs). Their results demonstrate that models like pix2pix and CycleGAN can effectively synthesize DWI maps, offering a promising method to augment clinical datasets and facilitate neuroimaging analyses reducing the need for time-consuming and resource-intensive DWI acquisitions.

## Conclusion and future directions

This Research Topic illustrates how artificial intelligence and machine learning, neuroinformatics and computational neuroscience can become a co-constitutive partner in the epistemic foundations of neuroscience and medicine.

**Figure 1:**
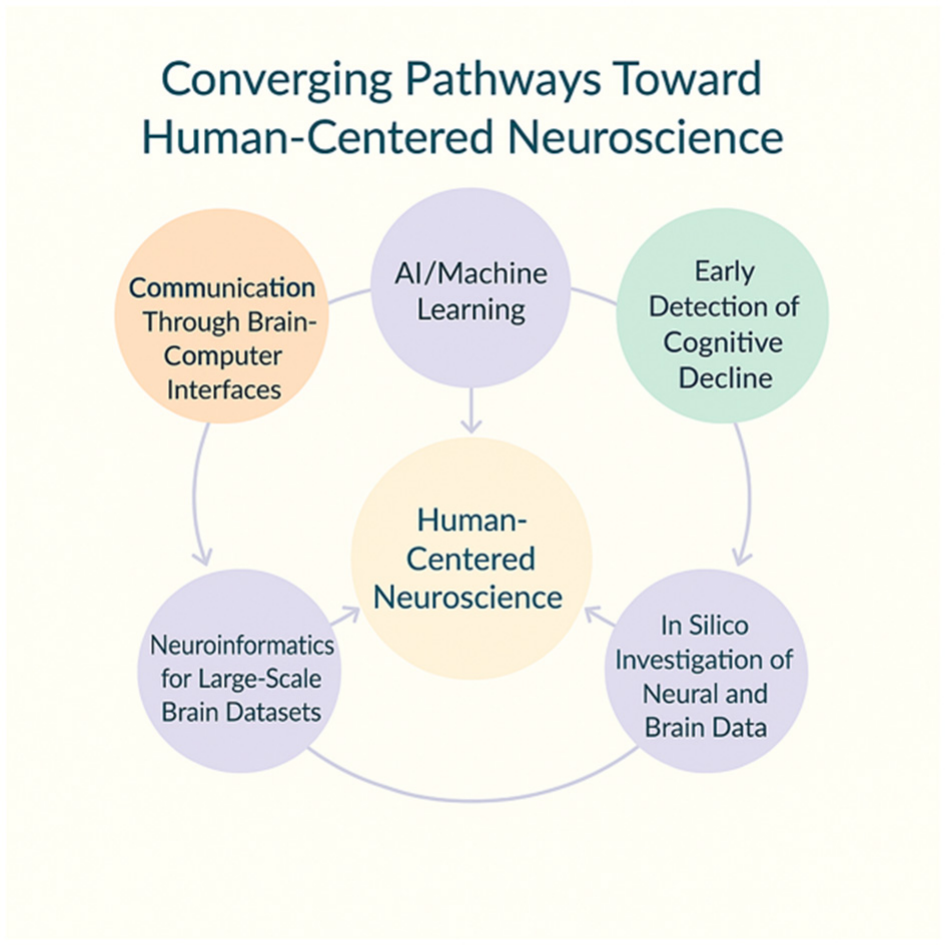
Converging pathways toward human-centered neuroscience. The diagram depicts converging pathways toward Human-Centered Neuroscience, emphasizing the synergy between computational and translational domains. Core computational nodes –AI/Machine Learning, Neuroinformatics for Large-Scale Brain Datasets, and In Silico Investigation of Neural and Brain Data –form an integrated analytical foundation driving innovation in neuroscience. These computational pathways connect to human-facing applications such as Communication Through Brain -Computer Interfaces and Early Detection of Cognitive Decline, illustrating how data-driven insights inform and refine clinical and societal outcomes. The circular design highlights the iterative flow of information, modeling, and feedback that anchors neuroscience in human relevance and ethical integration.

As illustrated in Figure 1, these domains are best understood not in isolation, but as nodes in a network where technological innovation and clinical practice converge. Such a perspective invites us to move beyond fragmented approaches, pointing toward a future in which translational, explainable, and equitable AI and computational tools become integral to how we see, measure, and treat the human brain and its disorders (Markram, 2013). Nevertheless, the future trajectory of this field demands sustained attention to validation, reliability, and ethical safeguards (Floridi et al., 2018). It is not sufficient to generate predictive accuracy; models must be tested across diverse populations, embedded in equitable systems of care, and rendered transparent to clinicians, patients, and caregivers. If achieved, this convergence will represent nothing less than a reconfiguration of neuroscience and psychiatry, where subjective evaluations from therapists and traditional clinical methods are used in synergy with predictive models, biomarkers and computational analyses. The women authors represented in this Research Topic exemplify how scientific innovation and gender diversity are both essential in the integration of neuroinformatics, machine learning, and brain medicine, and their contributions underscore the importance of diversity as a driver of equity, innovation, and scientific progress in tackling the complexity of the human brain.
